# Supplementary material for: End Sequence Analysis Toolkit (ESAT) expands the extractable information from single-cell RNA-seq data
Source: Genome Res. 2016 Oct;26(10):1397–410. doi: 10.1101/gr.207902.116 (PMC5052061; doi:10.1101/gr.207902.116)
Supplement: Supplemental Material [file supp_gr.207902.116_Supp_Materials.tar.gz › ESAT-esat_v0.1/doc/html/classumms_1_1core_1_1general_1_1_tabbed_reader.html]

ESAT: umms.core.general.TabbedReader Class Reference


ESAT


- Main Page
- Related Pages
- Packages
- Classes
- Files

- Class List
- Class Index
- Class Hierarchy
- Class Members

All Classes Namespaces Files Functions Variables Enumerator Pages

- umms
- core
- general
- TabbedReader

Classes |
Static Public Member Functions |
List of all members

umms.core.general.TabbedReader Class Reference

|  |  |
| --- | --- |
| Classes | |
| interface | Factory< T > |
|  | |
| class | **TabbedIterator< T >** |
|  | |

|  |  |
| --- | --- |
| Static Public Member Functions | |
| static< T > CloseableIterator< T > | read (File file, Class< T > clazz, TabbedReader.Factory<?extends T > factory, Predicate<?super T > filter, int skipRows) throws IOException |
|  | |
| static< T > CloseableIterator< T > | read (File file, Class< T > clazz, TabbedReader.Factory<?extends T > factory, Predicate<?super T > filter) throws IOException |
|  | |
| static< T > CloseableIterator< T > | read (File file, Class< T > clazz, TabbedReader.Factory<?extends T > factory, int skipRows) throws IOException |
|  | |
| static< T > CloseableIterator< T > | read (File file, Class< T > clazz, TabbedReader.Factory<?extends T > factory) throws IOException |
|  | |

## Member Function Documentation

|  |  |  |  |
| --- | --- | --- | --- |
| static <T> CloseableIterator<T> umms.core.general.TabbedReader.read | ( | File | *file*, |
|  |  | Class< T > | *clazz*, |
|  |  | TabbedReader.Factory<?extends T > | *factory*, |
|  |  | Predicate<?super T > | *filter*, |
|  |  | int | *skipRows* |
|  | ) |  | throws IOException |

static

Read an Annotation file line by line in a buffered format and return an iterator over the results. Should be refactored with the "load" function as appropriate ("load" can call this function and then add it to its Annotation list)

Parameters
:   |  |  |
    | --- | --- |
    | file |  |
    | clazz |  |
    | factory |  |
    | cs |  |
    | filter |  |
    | skipRows | number of rows to skip at the beginning of the file |

Returns

Here is the caller graph for this function:

|  |  |  |  |
| --- | --- | --- | --- |
| static <T> CloseableIterator<T> umms.core.general.TabbedReader.read | ( | File | *file*, |
|  |  | Class< T > | *clazz*, |
|  |  | TabbedReader.Factory<?extends T > | *factory*, |
|  |  | Predicate<?super T > | *filter* |
|  | ) |  | throws IOException |

static

Here is the call graph for this function:

|  |  |  |  |
| --- | --- | --- | --- |
| static <T> CloseableIterator<T> umms.core.general.TabbedReader.read | ( | File | *file*, |
|  |  | Class< T > | *clazz*, |
|  |  | TabbedReader.Factory<?extends T > | *factory*, |
|  |  | int | *skipRows* |
|  | ) |  | throws IOException |

static

Here is the call graph for this function:

|  |  |  |  |
| --- | --- | --- | --- |
| static <T> CloseableIterator<T> umms.core.general.TabbedReader.read | ( | File | *file*, |
|  |  | Class< T > | *clazz*, |
|  |  | TabbedReader.Factory<?extends T > | *factory* |
|  | ) |  | throws IOException |

static

Here is the call graph for this function:

---

The documentation for this class was generated from the following file:

- java/umms/core/general/TabbedReader.java


---

Generated on Fri Aug 15 2014 15:11:11 for ESAT by  

 1.8.7
